# Neonatal nurses’ performance in implementing the advancing newborn screening of critical congenital heart disease

**DOI:** 10.1186/s12912-025-04219-x

**Published:** 2025-12-30

**Authors:** Abdelaziz Hendy, Salma El-Sayed, Salah Mohamed Salah, Sawsan Abuhammad, Ahmed Hendy, Zainab Attia Abdallah

**Affiliations:** 1 Pediatric Nursing, Faculty of Nursing, Ain Shams University, Cairo, Egypt; 2 Faculty of Nursing, Damietta University, Damietta, Egypt; 3 Department of Nursing, College of Health Sciences, University of Sharjah, Sharjah, UAE; 4 Department of Maternal and Child Health, Faculty of Nursing, Jordan University of Science and Technology, Ar-Ramtha, Jordan; 5 Department of Computational Mathematics and Computer Science, Institute of Natural Sciences and Mathematics, Ural Federal University, Yekaterinburg, 620002 Russian Federation; 6 Department of Mechanics and Mathematics, Western Caspian University, Baku, 1001 Azerbaijan; 7 Community Health Nursing, Faculty of Nursing, Modern University for Technology and Information (MTI), Cairo, Egypt

**Keywords:** Critical congenital heart disease, Pulse oximetry, Neonatal nursing, Newborn screening, Attitude of health personnel, Cross-sectional studies

## Abstract

**Background:**

Critical congenital heart disease is a major cause of neonatal morbidity and mortality, requiring early detection and intervention. Pulse oximetry has been globally endorsed as a simple, cost-effective screening method. Despite its proven benefits, implementation in Egyptian neonatal care settings remains inconsistent. This study aimed to assess neonatal nurses’ knowledge, attitudes, and practices regarding the implementation of advanced newborn screening for Critical congenital heart disease using pulse oximetry across multiple hospitals in Egypt.

**Methods:**

A descriptive, cross-sectional study was conducted from December 2024 to April 2025 in nine NICUs across five governorates. A convenience sample of 279 neonatal nurses was recruited. Data were collected using a structured questionnaire assessing sociodemographic characteristics, knowledge, attitudes, and observational practice checklists. Descriptive statistics, correlation analysis, and multiple linear regression were used for data analysis.

**Results:**

The findings revealed unsatisfactory levels of knowledge (M = 3.56), negative attitudes (M = 12.55), and poor practice performance (M = 8.48). Strong positive correlations were found between knowledge, attitude, and practice (*r* = 0.59–0.68, *p* < .001). Regression analysis identified total knowledge, total attitude, neonatal experience, training attendance, and bachelor’s education as significant predictors of better practice (*p* < .01), while high nurse-to-patient ratios negatively impacted practice.

**Conclusion:**

Neonatal nurses demonstrated low proficiency in Critical congenital heart disease screening, largely due to educational and institutional limitations. Structured training, standardized protocols, and improved staffing ratios are essential to enhance screening performance and ensure early Critical congenital heart disease detection.

**Trial registration:**

Not applicable.

**Supplementary Information:**

The online version contains supplementary material available at 10.1186/s12912-025-04219-x.

## Background

Within the first year of life, a collection of structural cardiac defects known as critical congenital heart disease (CCHD) calls for either prompt medical or surgical treatment [1]. Reports from the World Health Organization indicate that, whereas in low-income countries it is twelve per thousand live births, in high-income countries, congenital heart disease (CHD) is eight per thousand live births [2]. As recorded in a study, catastrophic congenital heart disease (CHD) is rather common in Egypt [3].

Early diagnosis of these flaws is crucial to avoid hypoxia, hemodynamic impairment, and mortality. Increasingly used globally as a complement to routine newborn examination, pulse oximetry screening [4]. Studies in both high- and middle-income nations have shown its efficiency in spotting asymptomatic CCHD cases, enhancing early referral, and therefore improving outcomes [5, 6]. The American Academy of Pediatrics and the U.S. Department of Health and Human Services officially recommend universal CCHD screening in 2025 [7]. Many nations subsequently began to incorporate it into their national newborn screening initiatives.

Although newborn screening is somewhat common in Egypt, governmental hospitals and maternity clinics still hardly incorporate CCHD screening [8]. While limited implementation in tertiary care facilities and pilot studies shows promise, universal statewide acceptance remains erratic [1, 3]. Particularly for CCHD, the application of advanced newborn screening methods has significantly changed the duties of newborn nurses. These approaches, which use pulse oximetry as the main technique, have been rather popular in many hospitals since they help to improve early CCHD detection and therapy [5, 9]. Through screenings, education, and the preservation of continuity of care, neonatal nurses play a crucial role in this process. This increased responsibility requires nurses to be thoroughly aware of screening procedures and work closely with multidisciplinary teams to improve outcomes for children with CCHD [10].

Neonatal nurses are tasked with doing pulse oximetry assessments, interpreting data, and documenting results [11]. This action requires a thorough understanding of the screening process and the capacity to identify positive results that warrant further evaluation [12]. Nurses are essential in educating parents on the importance of CCHD screening and the implications of the results. This includes providing information on follow-up care and the potential need for further diagnostic procedures, such as echocardiograms [13, 14].

Combining a pulse oximeter with cardiac auscultation significantly improved the diagnosis rate of severe congenital heart disease in the early newborn period, according to a multicenter observational screening study conducted over 15 Shanghai hospitals [15]. Including 30,000 women and newborns, the Tanzania prospective cohort research looked at the use of pulse oximetry for the diagnosis of critical congenital heart disease (CCHD) in a resource-limited situation [16].

Several international studies highlight the importance of nursing competence in newborn screening implementation. For instance, Talus et al. (2023) found that appropriate training significantly increased nurses’ confidence and accuracy in neonatal screening. Similarly, Notarnicola et al. (2025) emphasized the need for institutional support, adequate staffing, and clear guidelines to ensure effective execution [17].

Neonatal nurses are essential components of multidisciplinary teams comprising neonatologists, pediatric cardiologists, obstetricians, and cardiothoracic surgeons. This team collaborates to deliver holistic care from diagnosis to treatment and recovery [12, 18]. In the delivery room, neonatal nurses collaborate with obstetricians and fetal cardiologists to prepare for and manage the immediate needs of newborns with CHD, ensuring that any necessary interventions are promptly administered [19]. Neonatal nurses operate within the cardiac critical care unit, collaborating with cardiac intensivists and other specialists to oversee the perioperative care of infants with CHD [20]. Prenatal detection of CHD allows for early planning and coordination among healthcare providers, including neonatal nurses, to optimize delivery and postnatal care strategies [21, 22].

Despite global endorsement of pulse oximetry, implementation of CCHD screening in Egyptian NICUs remains uneven and constrained by training gaps, workload, and unclear protocols. NICU nurses shoulder key responsibilities for screening, documentation, and parent education, yet their preparedness in this context has not been systematically evaluated in Egypt. Accordingly, we conducted a multicenter cross-sectional study to (1) quantify nurses’ knowledge, attitudes, and observed practice regarding advanced newborn screening for CCHD using pulse oximetry; (2) examine the interrelations among these domains; and (3) identify individual and organizational predictors such as education, training, experience, and nurse-to-patient ratios of screening performance to inform targeted training and protocol standardization.

## Methods

### Study design

We employed a multicenter cross-sectional design (December 2024–April 2025) in nine NICUs across five Egyptian governorates. This study was conducted to describe the current levels of neonatal nurses’ knowledge, attitudes, and observed screening practices for CCHD, and to examine how these variables are associated at a single point in time. The intent was to identify modifiable predictors such as training, education, neonatal experience, and nurse-to-patient ratio that may be linked to practice variation and could inform future implementation strategies. Also, the study is reported according to the guidelines for Strengthening the Reporting of Observational Studies in Epidemiology (STROBE). A cross-sectional approach was chosen because the objectives were descriptive and associational rather than causal, and because randomization or longitudinal follow-up was not feasible or ethical across multiple hospitals within the study window. We recognize that this design cannot establish temporality or causation, so we interpret findings as associations and propose prospective evaluations as a next step.

### Sampling and recruitment

A convenience sampling strategy was employed to recruit all eligible neonatal nurses who were actively providing direct care in the NICUs of participating hospitals during the designated data collection period. The inclusion criteria were registered nurses currently providing direct care to neonates in the NICU and having at least one year of NICU experience. Participation was voluntary, and only nurses who provided informed consent and expressed willingness to participate were included, in accordance with ethical guidelines. This approach aimed to capture as broad and representative a sample as possible within each hospital’s available workforce, while recognizing the inherent limitations of convenience sampling regarding potential selection bias and generalizability.

### Sample size

The sample size for this study was calculated using G*Power software for linear multiple regression with three predictors, a statistical power of 95%, and an alpha level of 0.05. The minimum required sample size was determined to be 254 participants. To account for a potential 10% attrition rate, an additional 25 nurses were included, bringing the final target sample size to approximately 279 participants.

### Setting

This multicenter study was conducted across 16 comprising both public and private hospitals distributed among five governorates in Egypt (Qalyubia, Sharkia, Menoufia, Cairo, and Giza), between December 2024 and April 2025. A total of 279 nurses recruited from these hospitals with site-specific numbers as follows: Qalyubia (48 nurses, three hospitals), Sharkia (39 nurses, three hospitals), Menoufia (30 nurses, two hospitals), Cairo (94 nurses, five hospitals), and Giza (68 nurses, three hospitals).

Participating NICUs were selected using pragmatic convenience sampling focused on units with high patient volume (60–90 admissions/month), sufficient incubator capacity (≥ 25 beds), and administrative approval. Selection prioritized government hospitals given their larger neonatal nurse workforce and critical care infrastructure, but private hospitals were also included to enhance sectoral and clinical diversity. The regional mix covers major urban centers and adjacent governorates to strengthen generalizability across varied care settings. However, selection was feasibility-based rather than probabilistic, and may introduce selection bias; as such, findings primarily apply to occupied NICU environments with similar institutional characteristics in the included regions.

### Data collection procedures and study instrument

A self-administered questionnaire was created by the researcher in Arabic following a survey of pertinent and contemporary literature. An English language version has been submitted as an ancillary file. It comprises the subsequent components:

### Part I: Sociodemographic and professional characteristics

The sociodemographic characteristics of the study participants included age, sex, Nursing Experience (Years), NICU Experience (Years), attendance at Training courses about CHD, Education Level, and Nurse-Patient Ratio.

### Part II: Knowledge (10 items)

This part was concerned with assessing nurses’ knowledge regarding implementing the advancing newborn screening of critical congenital heart disease; this tool was adapted from Ryan et al. [23] with minor contextualization to Egyptian NICU practice (Arabic translation/back-translation; expert review). The final instrument comprised 10 closed-ended items covering key aspects such as appropriate probe placement, timing of measurements, and interpretation of pulse oximetry results within the CCHD algorithm. Each item was scored 1 for a correct answer and 0 for an incorrect or “don’t know” response, yielding a total score from 0 to 10, with scores ≥ 80% of the maximum classified as satisfactory knowledge [24]. Internal consistency in the current study was acceptable (Cronbach’s alpha = 0.817).

### Tool (II): An observational checklist of the nurse’s practice

This part focused on evaluating the practical nursing implementation of advanced newborn screening of critical congenital heart disease. It was constructed by the researchers after comprehensively examining the pertinent literature [7, 23, 25, 26]. The tool comprised 22 items divided into five key domains: Preparation (three items), Performing the Pulse Oximetry (four items), Documentation (six items), Following the Screening Algorithm and Actions on Abnormal Results (five items), and Parent Education (four items), see supplementary file.

Each item was evaluated as either correctly performed (scored as “1”) or incorrectly/not performed (scored as “0”). The total score on the nurses’ practice checklist 22 was categorized as follows: a score < 80% was considered unsatisfactory, and a score ≥ 80% was considered satisfactory [24]. Internal consistency (current study): α = 0.886.

### Tool (III): Nurses’ attitude assessment

The researcher designed the assessment tool based on related literature [27] and [25] to evaluate nurses’ attitudes regarding implementing the advancing newborn screening of critical congenital heart disease and tool was refined by a five-member panel (two neonatal nursing faculty, one neonatologist, one quality officer, one charge nurse) for relevance and clarity.

The survey included 10 items on three domains as Attitude Toward Pulse Oximetry Screening for CCHD (3 items), Attitude Toward Following Clinical Guidelines (4 items), and Attitude Toward Patient Safety or New Interventions (3 items), see supplementary file. Participants provided responses using a 5-point Likert scale. The grading system was categorized as follows: (1) Strongly Disagree to (5) Strongly Agree. A score ≥ 80% reflects a positive attitude, while a score < 80% reflects a negative attitude [24]. Internal consistency (current study): α = 0.803.

### Procedure

After acquiring the requisite authorization from the respective hospitals and the neonatal intensive care unit, the ‘in the field’ work was carried out over a period spanning three months, from December 2024 to April 2025. The investigators initiated the process by informing the nursing personnel about the rationale for the study, and its aims and scope to ensure their collaboration. The authors did make it clear to the nursing staff that the feedback they provided was to be kept confidential, and the information was going to be treated solely for research purposes, focusing on data protection.

The CCHD screening protocol in the participating NICUs followed international recommendations, with pulse oximetry performed between 24 and 48 h after birth (or earlier if discharge occurred sooner) using pre-ductal (right hand) and post-ductal (either foot) measurements. A screen was considered failed if the oxygen saturation was less than 90% in either limb at any measurement, or if saturations remained below 95% in both limbs or differed by more than 3% between the right hand and foot on three consecutive readings obtained at least one hour apart. Infants with a failed screen were evaluated urgently by a physician and referred for echocardiography when no alternative cause of hypoxemia was identified, in line with contemporary CCHD screening guidance [20].

In these NICUs, pulse-oximetry screening was routinely performed for clinically stable term and late-preterm newborns (gestational age ≥ 34 weeks) who were not already diagnosed with major congenital heart disease and were cared for in the well-baby nursery or NICU prior to discharge.

### Checklist Preparation

The steps to create the checklist for pulse oximetry screening were systematic and based on proven medical practices. To assist the nurses, an initial thorough collection of relevant clinical protocols, including the National Screening Guidelines for Critical Congenital Heart Disease, was synthesized to ensure all instructional details necessary for nursing compliance were included. The checklist items were derived from national and international CCHD screening guidelines and prior implementation studies on pulse-oximetry screening [7, 12, 18, 20, 24].

Considering these guidelines, initial checklist items were noted and included probe placement, reading times, weighing outcomes, and responding to any flagged results. A working group including clinical nurse specialists, neonatal nurse practitioners, pediatricians, quality improvement personnel, and clinical educators was assembled to analyze the draft for clinical validity and completeness. Documents were fine-tuned to ensure precision while meeting checklist objectives and being simple to implement in everyday clinical work. User-friendly tables were created with clearly defined stages of care, including preparation, measurement, documentation, interpretation, and communication, including parent education. The checklist was evaluated for preliminary validation through peer review and pilot evaluation in a single clinical unit to evaluate practicality and usability.

### Observer training and standardization

Two data collectors, neonatal nurses with at least five years of NICU experience completed structured training before fieldwork. They reviewed a written standard operating procedure (SOP) that defined every checklist item and scoring rule, calibrated on five pilot videos and three live mock observations, and were certified after reaching at least 90% agreement with a senior reviewer. During data collection, both followed the same SOP and item-by-item decision rules. For operational reasons, one trained observer rated each shift at each site; inter-rater reliability was not estimated and is acknowledged as a study limitation.

In the accuracy and effectiveness of a clinical checklist, like a pulse oximetry screening in newborns, a thorough validation process was followed. Content validity was verified by a panel of five clinicians who reviewed checklist items. They rated each item using a 4-point relevance scale. The Content Validity Index (CVI) of the checklist’s items (I-CVI) was from 0.80 to 1.00, and the Scale-level CVI (S-CVI) was 0.92.

These results suggested strong content relevance across the tool. The checklist’s face validity was established by ten bedside nurses who reviewed the checklist’s wording and its use in clinical practice. Nurse respondents’ 90% said that the checklist was easy to integrate into their practice. Criterion validity was determined by comparison of checklist results with the counter’s expert observations and audits of the medical records. These found 96% sensitivity and 91% specificity in identifying protocol deviations. All these results together show the checklist is valid and reliable, reinforcing the case for its use in compliance monitoring with pulse oximetry screening protocols.

A pilot study involving 28 nurses (10% of the total sample size) was performed to test the clarity, applicability, relevance, and feasibility of the tools and to determine the time needed for data collection. After analyzing the pilot study’s results, the necessary modifications were made. Finally, the nurses involved in the pilot study were subsequently excluded from the study sample.

The data were collected twice a week on Saturdays and Tuesdays during both morning and afternoon shifts at the specified settings until the required sample size was achieved. These shifts followed the same nurse-to-patient staffing model and applied the same screening protocol; night shifts were not sampled because new screenings were rarely initiated overnight, and staffing patterns differed. Each participant underwent a 15-minute individual interview using the nurses’ knowledge assessment and Nurses’ Attitude Assessment tool. Additionally, nurses were observed by the researchers and assessed while performing nursing care for neonates throughout their shifts using observational checklists, which the researchers completed.

### Factor analysis for practice and attitude tools

To establish the construct validity and reliability of the study instruments, exploratory factor analysis was conducted for both the practice and attitude tools. The practice checklist, comprising twenty-two items, was analyzed using principal component extraction with varimax rotation. Sampling adequacy was confirmed (KMO = 0.872) and Bartlett’s test of sphericity was significant (χ² = 1984.56, *p* < .001). Five distinct factors were extracted, reflecting the theoretical domains of preparation, performing, documentation, following the screening algorithm, and parent education. These components explained 72.4% of the total variance, with all items showing loadings above 0.55. The internal consistency for each domain was robust, with Cronbach’s alpha values of 0.81 for preparation, 0.84 for performing, 0.86 for documentation, 0.89 for following the screening algorithm, and 0.83 for parent education indicating strong reliability and coherence across all domains.

Similarly, the ten-item attitude scale was examined using principal axis factoring with varimax rotation to verify its underlying structure. The KMO value (0.814) and Bartlett’s test (χ² = 596.22, *p* < 0.001) confirmed the adequacy of the data for factor analysis. Three factors were extracted in alignment with the conceptual framework attitude toward pulse oximetry screening, adherence to clinical guidelines, and openness to patient safety or new interventions explaining 68.9% of the total variance. Factor loadings ranged from 0.57 to 0.84, and reliability analysis revealed Cronbach’s alpha values of 0.79, 0.82, and 0.80 for the respective domains. These findings validate the construct integrity of the tools and confirm their suitability for accurately assessing neonatal nurses’ practice and attitudes toward advanced newborn screening for critical congenital heart disease.

### Statistical analysis

The data were analyzed utilizing SPSS version 26.0. Descriptive statistics were employed to delineate the general characteristics. Pearson’s correlation was utilized to evaluate the strength and direction of correlations between knowledge, attitudes, and practices. Multiple linear regression with 95% confidence intervals was then applied to identify predictors of total practice, with model diagnostics (residual plots, variance inflation factors) supporting acceptable fit and minimal multicollinearity. Multiple linear regression analysis showed several significant predictors of the dependent variable “Total Practice” classified as independent factors. The model comprises the following independent variables: age, years of experience, gender, educational attainment, training courses, nurse-to-neonate ratio, overall knowledge, and overall attitude. The normality of continuous variables was assessed with the Shapiro-Wilk test. For Total practice (W = 0.987, *p* = .072), the data were normally distributed, justifying the use of parametric tests in later analyses. All Variance Inflation Factor (VIF) values were less than 3, so there were no issues of multicollinearity among the predictors. The instrument’s reliability has been assessed using Cronbach’s alpha test which produced the following outcomes: knowledge (α = 0.817), practice (α 0.886), and attitude (α 0.803). *P* < 0.05 was considered to indicate significance.

Consistent with previous nursing KAP studies in Egypt and comparable contexts, scores ≥ 80% of the maximum possible were classified as ‘satisfactory’ knowledge or practice and ‘positive’ attitude, whereas scores < 80% were considered unsatisfactory or negative.

## Results

**Table 1:** Characteristics of studied nurses (n=279)

Variable	Categories	N	%
Age	Mean (SD)	33.43	6.84
Nursing Experience (Years)	Mean (SD)	10.56	5.79
NICU Experience (Years)	Mean (SD)	5.60	2.77
Gender	Male	92	32.90
	Female	187	67.10
Attended Training	Yes	100	35.80
	No	179	64.20
Education Level	Bachelor	100	35.80
	Technical Health	140	50.20
	Diploma	39	14.00
Nurse-Patient Ratio	1 to 1	40	14.30
	1 to 2	95	34.10
	1 to 3	144	51.60

NICU (Neonatal Intensive Care Unit), SD (Standard Deviation)

Table 1 presents the sociodemographic and professional characteristics of the 279 nurses included in the study. The mean age was 33.43 years (SD = 6.84), and the average years of nursing experience was 10.56 years (SD = 5.79). The sample was predominantly female (67.1%). In terms of educational attainment, half of the nurses held a technical health certificate (50.2%). Notably, 64.2% of the nurses reported not attending training courses about the care of congenital heart diseases. Additionally, more than half of nurses were working under a 1:3 nurse-to-patient ratio (51.6%).

**Table 2:** Mean score of nurses’ knowledge, attitude, and practice regarding advancing newborn screening of critical congenital heart disease (*n* = 279)

	Mean	Standard Deviation	Minimum	Maximum	Interpretation
Practice (Preparation)	1.58	0.49	1.0	2.0	Unsatisfactory
Practice (Performing)	1.59	0.49	1.0	2.0	Unsatisfactory
Practice (Documentation)	1.72	0.84	0.0	3.0	Unsatisfactory
Practice (Follow Algorithm)	2.68	0.73	2.0	4.0	Unsatisfactory
Practice (Parent Education)	0.90	0.29	0.0	1.0	Unsatisfactory
**Total practice updated**	8.48	1.61	4.0	12.0	Unsatisfactory
Attitude (CCHD)	3.35	0.52	3.0	5.0	Negative
Attitude (Guidelines)	5.72	0.79	5.0	8.0	Negative
Attitude (Safety)	3.47	0.58	3.0	5.0	Negative
**Total Attitude**	12.55	1.28	11.0	16.0	Negative
**Total Knowledge**	3.56	1.14	2.0	6.0	Unsatisfactory

CCHD **(**critical congenital heart disease)

**Fig. 1:**
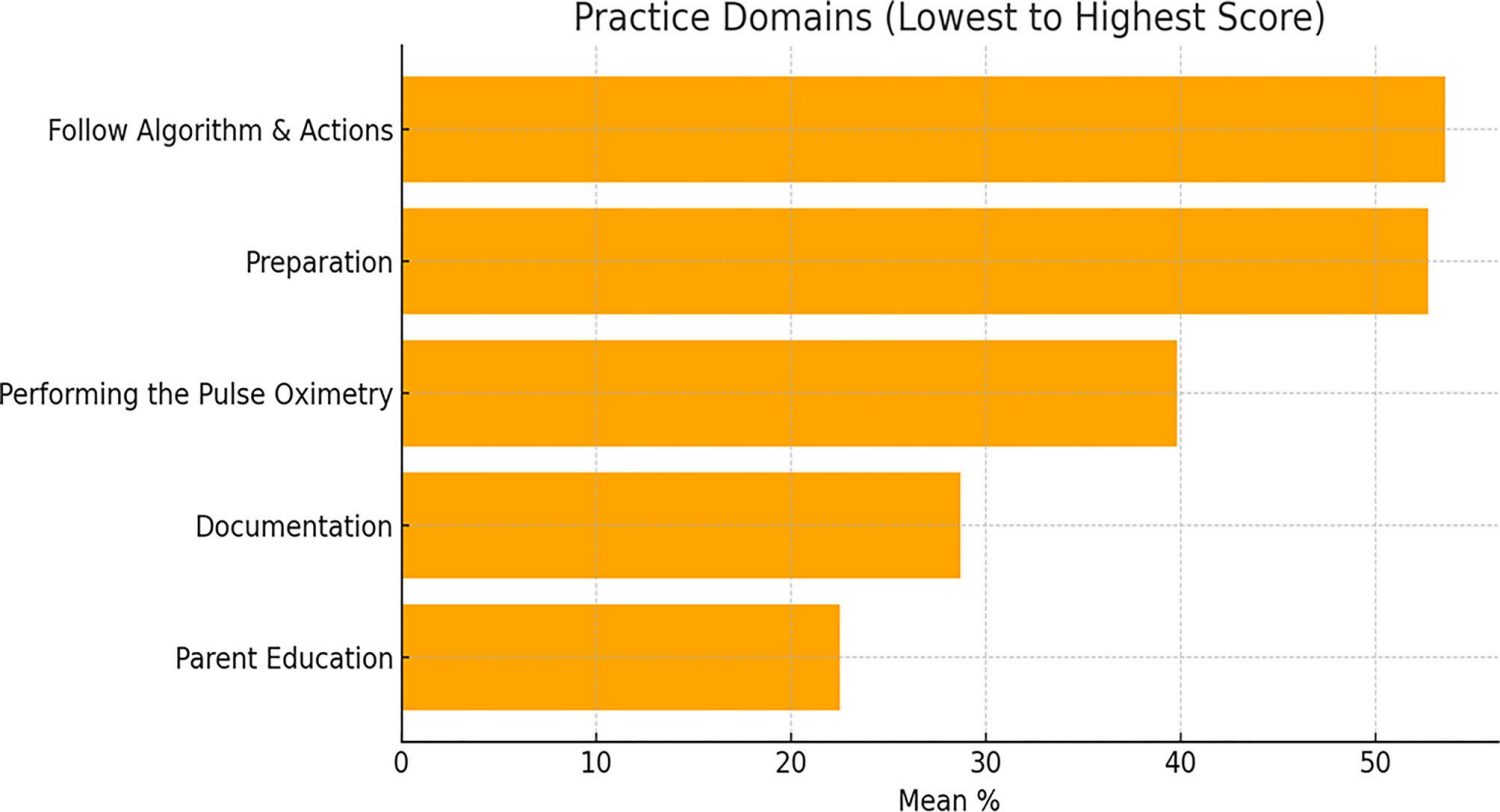
Mean percentage scores of practice domains ordered from lowest to highest

A descriptive analysis regarding the knowledge, attitude, and practice of the nurses showed that their performance perceptions concerning CCHD screening were below the expected level and were negative (see Table 2). The mean score for total knowledge was M = 3.56, SD = 1.14. Practice scores across all domains were similarly low. Specifically, spread over the pre-defined domains: Preparation (M = 1.58, SD = 0.49), Performing (M = 1.59, SD = 0.49), Documentation (M = 1.72, SD = 0.84), Following the Algorithm (M = 2.68, SD = 0.73), and Parent Education (M = 0.90, SD = 0.29), all categorized as unsatisfactory. These were further reinforced with attitude toward CCHD (M = 3.35, SD = 0.52), Guidelines (M = 5.72, SD = 0.79), and Safety (M = 3.47, SD = 0.58). In light of everything, the total attitude score was negative (M = 12.55, SD = 1.28) (Fig 1).

This figure displays the mean percentage scores of practice-based domains involved in pulse oximetry screening. While Follow Algorithm & Actions (53.6%) and Preparation (52.7%) were among the highest, other critical areas like Documentation (28.7%) and Parent Education (22.5%) were significantly lower.

**Fig. 2:**
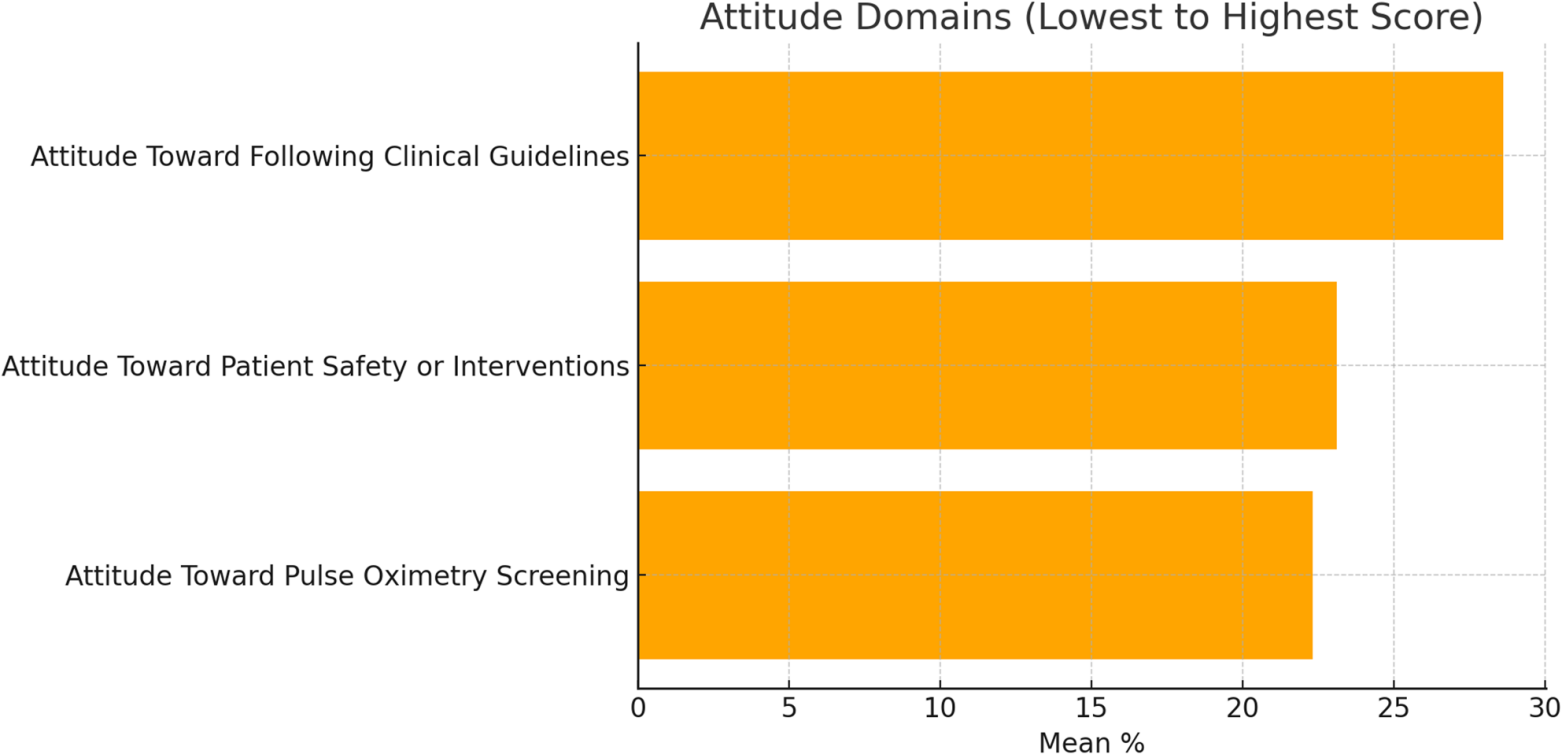
Mean percentage scores of attitude domains ordered from lowest to highest

This chart shows that all attitude-related domains scored below 30%, which reflects a lack of strongly positive attitudes towards pulse oximetry screening, patient safety, and adherence to the clinical practice guidelines. The lowest score, Pulse Oximetry Screening Attitude (22.3%), suggests a need to improve the way practitioners perceive and value the screening process (Fig. 2).

**Table 3:** Mean score of nurses’ knowledge, attitude, and practice regarding advancing newborn screening of critical congenital heart disease (*n* = 279)

Variable 1	Variable 2	Correlation (*r*)	*p*-value
Total Knowledge	Total practice	0.59	0.00
Total Knowledge	Total Attitude	0.68	0.00
Total practice	Total Attitude	0.61	0.00

Relationships among nurses’ knowledge, attitudes, and practice scores were analyzed utilizing a Pearson correlational analysis (refer to Table 3). The results demonstrated a moderate to strong positive connection between total knowledge and total practice, *r* = .59, *p* < .001, suggesting that increased information enhances practice. Total knowledge had a robust correlation with total attitude, *r* = .68, *p* < .001, indicating that increased information is likely to elicit more favorable views. A moderate positive connection was identified between overall attitude and total practice, *r* = .61, *p* < .001.

**Table 4:** Multiple linear regression analysis for nurses’ practice (*n* = 279)

Variable	Coefficient	Std Error	t-value	*p*-value	VIF
const	0.68	0.92	0.74	0.459	
Total Knowledge	0.31	0.10	3.00	0.003	2.80
Total Attitude	0.40	0.07	5.09	0.00	1.95
Age	0.01	0.01	1.30	0.194	1.03
Experience Nursing (Years)	0.006	0.01	0.44	0.66	1.23
Experience Neonatal (Years)	0.15	0.03	5.19	0.00	1.30
Attended Training (Yes)	1.09	0.18	6.05	0.00	1.45
Education (Bachelor)	0.45	0.16	2.78	0.006	1.18
Ratio (1 to 3)	-0.68	0.14	-4.71	0.00	1.02

F 54.09, *P* < .001, R: 0.785, R²: 0.616, Effect Size (Cohen’s f²): 1.603

Dependent variable: Nurses’ PracticePredictors: Total knowledge, Total Attitude, Experience Nursing (Years), Experience Neonatal (Years), Attended Training (Yes), Education (Bachelor), Ratio (1 to 3)

A multiple linear regression was performed to explore the predictors of nurses’ practice scores. The model was significant, F = 54.09, *p* < .001, with 61.6% of the variance in practice scores explained (R² = 0.616), which showed strong model fit (*R* = .785). The effect size was large (Cohen’s f² = 1.603), indicating a high impact of the predictors on the outcome variable. Among the predictors, total knowledge (β = 0.31, *p* = 0.003) and total attitude (β = 0.40, *p* < 0.001) were significant and positive, reinforcing the proposition that greater knowledge and more positive attitudes influence nursing practice. Also, neonatal experience (β = 0.15, *p* < 0.001), attendance to training (β = 1.09, *p* < 0.001), and possession of a bachelor’s degree (β = 0.45, *p* = .006) were positively associated with improved practice. In the other direction, a nurse-to-patient ratio of 1:3 showed a significant negative impact (β = –0.68, *p* < .001), indicating that higher patient loads inhibit the application of good practice standards. The participant’s age and years of working in nursing did not show any statistically significant results (*p* > 0.05). All VIF values were less than 3, so there were no issues of multicollinearity among the predictors. See Table 4.

## Discussion

The present study sought to investigate the degree of nurses’ knowledge, attitudes, and practices regarding the adoption of enhanced neonatal screening for serious congenital heart disease, along with the interrelationships among these variables. The results indicated that neonatal nurses exhibited predominantly inadequate levels of knowledge, attitude, and practice about the execution of advanced screening for CCHD. These results are consistent with Alsherif et al., who discovered that most nurses have inadequate knowledge [28]. A cross-sectional study including 189 nurse supervisors was conducted to validate methods for the implementation of pulse oximetry screening for CCHD, revealing a low awareness of critical screening thresholds among the supervisors [10]. While, in contrast, a study in Sri Lanka reported high levels of acceptable knowledge regarding pulse oximetry protocols [29].

These low levels can be attributable to a variety of critical factors. Initially, the study results confirm that there is a lack of education, as half of the nurses possess a technical health certificate. Additionally, the study results confirm that there is a shortage of training, as two-thirds of the nurses reported not attending training courses regarding the management of congenital heart diseases. Secondly, work-related factors: a nurse-to-patient ratio of 1:3 was observed in over half of the nurses. Finally, there are no established procedures. All these things make it harder to put CCHD screening programs into place, which are very important for finding and treating the illness in babies as soon as possible. These results show that nurses need to keep learning, so they know about the latest recommendations and best practices for CCHD screening.

These findings indicate the necessity for continuous education to ensure nurses remain informed about the newest recommendations and best practices for CCHD screening. Healthcare institutions implement several techniques to guarantee that nurses obtain prompt updates on the most recent standards for CCHD screening and optimal practices. The solutions encompass the formulation of uniform protocols, as evidenced by a study conducted in the USA [25], and the implementation of educational toolkits, given that nurses in this study indicated the lowest performance in the parent education subscale. Educational toolkits, like those created by the Children’s National Medical Center, offer evidence-based resources for the training of healthcare personnel, including nurses, on the most recent CCHD screening protocols [25]. A study in China sought to expand the understanding of neonatal congenital cardiac disease and indicated that substantial knowledge improvement was observed following standardized training on the disease and reported that significant knowledge gain occurred after standardized training in the [30]. Numerous studies demonstrate that instructional initiatives, such as online modules, videos, and in-service training, substantially enhance nurses’ knowledge and screening efficacy [23, 28, 31].

Moreover, using established techniques for CCHD screening, particularly in environments such as the NICU, ensures uniformity in practice. It profoundly impacts the practice of neonatal nurses by offering a systematic framework for implementation, assuring uniformity, and improving the quality of care. These procedures assist nurses in conducting screens, analyzing results, and instructing families, thereby enhancing early diagnosis and intervention for CCHD. The results of a review article support the claim that comprehension of the essential aspects of screening significance, screening techniques, current follow-up guidelines for positive outcomes, and the constraints of CCHD screening empowers nurses to advocate effectively for their patients, thereby improving outcomes for infants born with CCHD through early detection before discharge [12]. A multimodal quality improvement program at a children’s hospital in Washington revealed that the adoption of standardized processes is associated with a significant reduction in documentation mistakes and protocol violations, leading to more reliable and accurate screening results [32].

Among the practice subscales, parent education and documentation had the lowest performance; however, adherence to the screening methodology was comparatively superior. Attitudinally, apprehensions regarding safety and the significance of CCHD screening were markedly minimal. Numerous investigations have highlighted distinct deficiencies in understanding, especially for the interpretation of screening results [29, 33]. Conversely, favorable attitudes on the significance of CCHD screening are frequently documented, with nurses acknowledging the benefits of early detection and demonstrating a readiness to participate in screening activities [34, 35].

This discrepancy can be elucidated by many contextual considerations. In numerous low- to middle-income nations such as Egypt, healthcare systems frequently have substantial resource limitations, including personnel deficits and elevated patient volumes, which may restrict the time allocated for thorough documentation and parental involvement. As a result, nurses may prioritize technical and time-sensitive elements of care, such as adhering to the screening algorithm, over supportive tasks like educating parents or meticulously recording procedures.

Moreover, deficiencies in nursing education and training may exacerbate this disparity, as clinical courses frequently prioritize procedural skills over communication and documentation procedures. Cultural variables may influence the situation; in certain contexts, parental engagement in newborn care is restricted, and it is often presumed that physicians, rather than nurses, are tasked with conveying information to families. The lack of defined institutional norms or resources for parent education and documentation may impede consistent practice in these domains.

Notwithstanding these limitations, the study observed that nurses had few attitudinal apprehensions concerning the safety or efficacy of CCHD screening, reflecting an overall favorable opinion of the program. This indicates that the diminished performance in specific subscales is not attributable to insufficient dedication but rather signifies institutional and logistical obstacles that must be resolved to provide a more thorough execution of newborn screening activities.

The positive correlations among knowledge, attitude, and practice highlight the interrelation of these elements. The robust correlation between knowledge and attitude indicates that enhancing educational interventions could yield twofold advantages in both practice and perception. This corresponds with prior data suggesting that knowledgeable healthcare professionals typically have superior clinical actions and attitudes. The evidence indicates a positive link whereby more knowledge promotes good attitudes, therefore improving adherence to screening measures [23, 34, 36]. Research indicates that nurses possessing elevated knowledge scores are more inclined to conduct tests accurately and promote the significance of screening [34, 36]. This triumvirate is essential for attaining high-quality screening and early diagnosis of CCHD [23].

The link among knowledge, attitude, and practice is intricate. Although elevated knowledge and favorable attitudes often enhance screening performance, they are insufficient on their own to ensure effective practice. Consequently, comprehensive interventions targeting instructional content, attitudinal support, workflow integration, and systemic infrastructure are essential to improve neonatal screening efficacy. Ultimately, enhancing nurses’ competencies and cultivating supportive environments will facilitate the early detection of CCHD, improve patient outcomes, and diminish morbidity and mortality linked to undetected heart abnormalities in neonates.

In this research, we employed a regression model to ascertain the contribution of these characteristics to nursing practices. Total knowledge and total attitude emerged as significant and positive predictors, substantiating the assertion that enhanced knowledge and favorable attitudes impact nursing practice. The regression model emphasized the beneficial impacts of neonatal experience, specialized training, and a bachelor’s degree on nursing practice. A nurse-to-patient ratio of 1:3 adversely affected practice, highlighting structural challenges such as staffing shortages that could hinder the application of best practices, regardless of individual expertise or attitude.

### Implications of practice

Pulse oximetry as a screening method for CCHD requires the creation and application of comprehensive training programs. Employers need to focus on parent communication and education training as part of ongoing professional development. Having unambiguous screening instructions, reasonable nurse-to-patient ratios, and current policy and procedure manuals greatly increases organizational screening outcomes. Provision of educational toolkits and simulation-based teaching materials can increase nurses’ effectiveness and self-assurance, enabling them to provide better patient care.

### Limitations

Because it is cross-sectional, we cannot infer causality and the timing of relationships is uncertain, so links between knowledge, attitude, and practice should not be read as directional. Convenience sampling may introduce selection bias and reduces generalizability beyond the participating NICUs. Self-reported knowledge and attitudes, together with single-rater observations of practice, can introduce measurement and observer bias; inter-rater reliability was not assessed. Despite adjustment for key covariates, residual confounding likely remains. We also did not account for clustering by site, which may affect standard errors. Future studies should use prospective or longitudinal designs, dual-observer reliability checks, and multi-level models to strengthen inference.

## Conclusion

This study revealed that neonatal nurses in Egypt demonstrate unsatisfactory levels of knowledge, attitudes, and practices regarding the implementation of advanced newborn screening for CCHD. Despite the recognized importance of pulse oximetry as a non-invasive and effective tool for early CCHD detection, gaps in education, training, and institutional support persist. The findings highlight a strong and statistically significant correlation between nurses’ knowledge, attitudes, and their clinical practice performance, emphasizing the crucial role of structured education and favorable work environments in achieving optimal screening outcomes. Addressing these gaps is essential to ensuring early identification, timely intervention, and improved survival rates for infants with CCHD.

## Supplementary Information

Below is the link to the electronic supplementary material.

Supplementary Material 1

## Data Availability

The data are provided within the manuscript and supplementary file.

## Abbreviations

CCHD: Critical Congenital Heart Disease
CHD: Congenital Heart Disease
NICU: Neonatal Intensive Care Unit
LE: Life Expectancy
LD: Life Disparity
AAP: American Academy of Pediatrics
CDC: Centers for Disease Control and Prevention
KAP: Knowledge, Attitudes, and Practices
IRB: Institutional Review Board
SPSS: Statistical Package for the Social Sciences
CVI: Content Validity Index
I-CVI: Item-level Content Validity Index
S-CVI: Scale-level Content Validity Index
VIF: Variance Inflation Factor
SD: Standard Deviation
DF: Degrees of Freedom
CI: Confidence Interval
