# Supplementary material for: Neonatal nurses’ performance in implementing the advancing newborn screening of critical congenital heart disease
Source: BMC Nurs. 2025 Dec 30;25:93. doi: 10.1186/s12912-025-04219-x (PMC12849507; doi:10.1186/s12912-025-04219-x)
Supplement: Supplementary file 1 — Supplementary Material 1 [file 12912_2025_4219_MOESM1_ESM.docx]

**Tool**

# Pulse Oximetry Screening Checklist for Nurses (CCHD Screening)

| **Steps** | **Checklist Item** |
| --- | --- |
| Preparation |  Confirm newborn is **≥24 hours old**   Gather and check pulse oximeter and neonatal probes   Ensure newborn is **calm, warm, and settled** |
| Performing the Pulse Oximetry |  Place probe on **right hand** (pre-ductal site)   Record **oxygen saturation from right hand**   Place probe on **right foot** (post-ductal site)   Record **oxygen saturation from right foot** |
| Documentation |  Document **date and time** of each reading   Document **location of probes** (right hand and right foot)   Document **oxygen saturation values** from both sites   Document if there is a **≥3% difference** between readings   Document whether the **screening passed or failed**   If screening failed:   - Document that **protocol for failed screening** was followed - Document **repeat readings** (if applicable) - Document **provider notification** - Document any **follow-up tests or actions** |
| Follow Screening Algorithm  And Actions on Abnormal Results |  Review results against screening criteria:   - **<90%** in either extremity = **Immediate Fail** - **90–94% in both extremities OR >3% difference** = **Repeat in 1 hour (up to 3 times)**    Proceed based on algorithm outcomes   Notify **pediatrician or cardiologist**   Arrange **diagnostic evaluation** (e.g., echocardiogram)   Continue **monitoring and documentation** |
|  |  |
| Parent Education |  Explain **what pulse oximetry screening is**   Discuss **possible results** and next steps   Clarify potential for **false positives**   Instruct on signs/symptoms of **CHD** to watch for |

**Attitude scale:**

Each item is intended for a **5-point Likert scale**: **(1) Strongly Disagree** to **(5) Strongly Agree**

**Domain 1: Attitude Toward Pulse Oximetry Screening for CCHD:**

1. Pulse oximetry screening is a valuable tool for early detection of critical congenital heart disease (CCHD) in newborns.
2. Performing pulse oximetry as part of routine newborn care improves patient outcomes.
3. I believe pulse oximetry screening adds unnecessary workload to my shift. (Reverse-coded)

**Domain 2: Attitude Toward Following Clinical Guidelines**:

1. Adhering to clinical screening protocols ensures safe and consistent care for newborns.
2. I feel confident interpreting and applying clinical guidelines in my daily practice.
3. Following guidelines helps reduce errors and improves care quality.
4. Clinical guidelines limit my professional judgment in newborn care. (Reverse-coded)

**Domain 3: Attitude Toward Patient Safety or New Interventions**:

1. I am open to adopting new clinical interventions if they improve patient safety.
2. Patient safety should always be prioritized, even when it requires changes to routine practices.
3. Introducing new interventions often disrupts workflow without real benefits. (Reverse-coded)
